# Meta-analysis of prognostic factors in patients with knee arthroplasty

**DOI:** 10.3389/fsurg.2025.1584238

**Published:** 2025-07-18

**Authors:** Fengying Guo, Xiaoxia Shi, Honghui Song, Shendong Wang

**Affiliations:** Department of Orthopaedics, Second Affiliated Hospital of Soochow University, Suzhou, Jiangsu, China

**Keywords:** knee replacement, prognosis, influencing factors, meta-analysis, knee osteoarthritis

## Abstract

**Objective:**

To identify specific factors predicting functional outcomes, pain reduction, and patient satisfaction following knee arthroplasty through systematic review and meta-analysis.

**Methods:**

A comprehensive search of multiple databases (Pubmed, Embase, OVID, Medline, Cochrane Library, CNKI, Wanfang, VIP) was conducted for studies published from database inception to December 2024. Studies reporting associations between preoperative factors and standardized outcomes after knee arthroplasty were included. Two reviewers independently screened articles, extracted data, and assessed study quality using modified Jadad scale for randomized trials and MINORS for non-randomized studies. Random-effects meta-analyses were performed for pain duration and red blood cell distribution width (RDW), with meta-regression to assess their prognostic value for functional outcomes measured by standardized knee scores. Heterogeneity was assessed using I^2^ statistics, and publication bias was evaluated using Egger's and Begg's tests.

**Results:**

Eight studies were included in the final analysis: Four studies examining pain duration (*n* = 576 patients) and four studies examining RDW (*n* = 612 patients) met inclusion criteria. Significant heterogeneity was observed in both analyses (I^2^ = 87% and I^2^ = 91%, respectively, *p* < 0.01). Meta-regression revealed that shorter pain duration (<3 years) was significantly associated with better functional outcomes at 12-month follow-up [Weighted Mean Difference (WMD) = −4.532, 95%CI = (−6.439,−2.626), *p* < 0.001]. Normal preoperative RDW values (11.5–14.5%) were also significantly associated with improved functional outcomes [WMD = −1.742, 95%CI = (−2.371,−1.114), *p* < 0.001]. Subgroup analyses indicated that the predictive value of these factors was consistent across different surgical techniques (*p* = 0.42). Publication bias assessment showed no significant bias (Egger's test *p* = 0.2094, Begg's test *p* = 0.0833). The high heterogeneity limits the direct clinical application of these pooled estimates and necessitates cautious interpretation.

**Conclusion:**

This meta-analysis identified shorter preoperative pain duration and normal RDW values as independent predictors of better functional outcomes following knee arthroplasty. However, the small number of included studies and high heterogeneity observed warrant cautious interpretation of these findings. These findings can help clinicians identify patients at risk of suboptimal outcomes and potentially guide personalized perioperative interventions. Further research is needed to establish optimal cutoff values and to evaluate the combined predictive power of these factors in clinical practice.

## 1 Introduction

Knee osteoarthritis (KOA) is a common degenerative condition in elderly populations that significantly impacts quality of life through pain, stiffness, and functional limitation. For end-stage KOA (Kellgren-Lawrence grades III-IV), characterized by substantial joint space narrowing and osteophyte formation, conservative treatments typically provide inadequate relief (1). In these cases, surgical intervention through knee arthroplasty becomes the primary treatment option.

Total knee arthroplasty (TKA) represents one of the most successful orthopedic interventions for end-stage KOA, with reported satisfaction rates between 75% and 97% (2). Despite this success, approximately 15%–25% of patients experience suboptimal outcomes, reporting persistent pain, functional limitations, or dissatisfaction following surgery. Identifying preoperative factors that predict these outcomes would enable better patient selection, expectation management, and potentially guide personalized interventions to improve results.

Previous research has identified multiple potential predictors of TKA outcomes, including patient factors (age, gender, BMI, nutritional status, psychological factors, pain duration, preoperative function), surgical factors (anesthesia type, surgical technique, implant selection), and rehabilitation protocols (3, 10). Santaguida et al. conducted a comprehensive systematic review examining patient characteristics affecting the prognosis of total hip and knee joint arthroplasty, highlighting the importance of identifying modifiable risk factors (10). While these studies provide valuable insights, many have produced conflicting results or identified associations too weak for clinical application. Additionally, while demographic and surgical factors have been extensively studied, biological markers that might predict outcomes have received comparatively less attention.

Among potential biological predictors, inflammatory markers have shown promise in predicting TKA outcomes. The red blood cell distribution width (RDW), an inexpensive and routinely measured parameter, has emerged as a potential prognostic indicator. Originally used to classify anemias, elevated RDW has been associated with inflammation, cardiovascular disease, and poor outcomes in various medical conditions (4). In the context of TKA, preliminary studies suggest that abnormal RDW may predict complications including infection, venous thromboembolism, and potentially functional outcomes (5, 14). Recent work by Garval et al. further identified multiple prognostic factors of knee pain and function 12 months after TKA in a large prospective cohort study (14).

Similarly, preoperative pain duration has been proposed as a predictor of TKA outcomes, with some evidence suggesting that chronic, long-standing pain may be associated with central sensitization and poorer response to surgery. However, these factors have not been systematically evaluated through meta-analysis.

The present study aims to systematically review and meta-analyze the available evidence on preoperative factors that predict functional outcomes after knee arthroplasty, with specific focus on pain duration and RDW. By identifying reliable predictors of outcomes, we hope to provide clinicians with tools to better select candidates for surgery, manage expectations, and potentially develop interventions to improve results in higher-risk patients.

## 2 Data and methods

### 2.1 Study registration and protocol

This systematic review and meta-analysis was conducted in accordance with the Preferred Reporting Items for Systematic Reviews and Meta-Analyses (PRISMA) guidelines. The study protocol, including search strategy, inclusion/exclusion criteria, and analytical methods, was established prior to the literature search.

### 2.2 Literature search strategy

A comprehensive search was conducted in electronic databases including PubMed, Embase, OVID, Medline, Cochrane Library, CNKI, Wanfang, and VIP from their inception to December 2024. Additionally, ClinicalTrials.gov and reference lists of relevant studies were searched to identify additional eligible studies.

The search was conducted using combinations of the following keywords: “knee arthroplasty,” “knee replacement,” “TKA,” “prognosis,” “outcome,” “prediction,” “functional recovery,” “pain duration,” “red blood cell distribution width,” and “RDW.” Both English and Chinese language publications were considered for inclusion.

### 2.3 Eligibility criteria

Studies were selected based on the following criteria:

Inclusion criteria:

1. Study design: Randomized controlled trials or observational studies (prospective or retrospective cohort studies)
2. Population: Adult patients (≥18 years) undergoing primary knee arthroplasty
3. Predictors: Studies reporting on preoperative pain duration and/or red blood cell distribution width (RDW)
4. Outcomes: Functional outcomes measured by validated knee scores (e.g., KSS, WOMAC, Oxford Knee Score), pain scores, or patient satisfaction at a minimum follow-up of 6 months
5. Statistical reporting: Studies providing sufficient data for effect size calculation (means, standard deviations, odds ratios, or hazard ratios with 95% confidence intervals)

Exclusion criteria

1. Duplicate publications, conference abstracts without full-text, or studies with insufficient data for analysis
2. Studies focusing on revision knee arthroplasty
3. Case reports, reviews, or animal studies
4. Studies with high risk of bias or methodological quality score below the predetermined threshold

### 2.4 Study selection process

**Figure 1:**
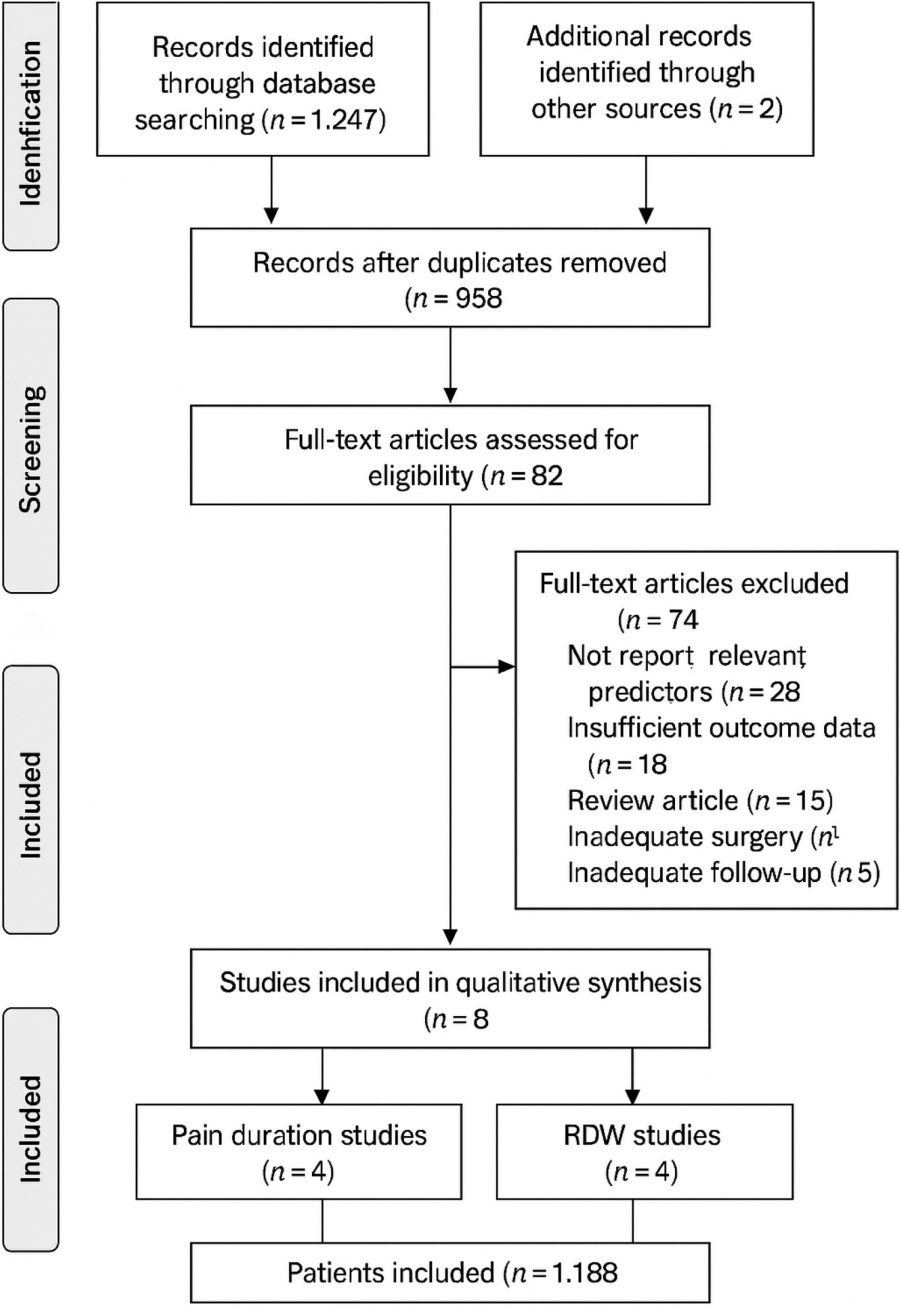
PRISMA flow diagram showing the study selection process.

Two reviewers independently screened titles and abstracts of all identified records for potential eligibility. Full texts of potentially eligible studies were then assessed independently by the same reviewers. Disagreements were resolved through discussion or, if necessary, consultation with a third reviewer. The selection process was documented using a PRISMA flow diagram (Figure 1).

### 2.5 Data extraction

A standardized data extraction form was used to collect the following information: first author, publication year, study design, sample size, patient demographics (age, sex, BMI), surgical procedure details, preoperative pain duration (in months or years), preoperative RDW values, outcome measures, follow-up duration, and statistical results. Specific definitions of pain duration categories varied across studies: three studies defined short duration as <3 years while one study used <2 years; all studies defined normal RDW as 11.5–14.5%. For studies with multiple follow-up points, data from the latest follow-up were extracted. Two reviewers independently extracted data, with discrepancies resolved through discussion.

### 2.6 Quality assessment

The methodological quality of included randomized controlled trials was assessed using the modified Jadad scale, with studies scoring ≥3 considered high quality. For non-randomized studies, the Methodological Index for Non-Randomized Studies (MINORS) was used, with scores ≥16 for comparative studies and ≥12 for non-comparative studies considered high quality. Quality assessment was performed independently by two reviewers, with disagreements resolved through discussion or third-party arbitration.

### 2.7 Statistical analysis

All analyses were performed using Review Manager 5.4.1 (RevMan 5.4.1, Cochrane Collaboration) and Stata 16.0 (StataCorp, College Station, TX). For continuous outcomes, weighted mean differences (WMD) with 95% confidence intervals (CI) were calculated. For dichotomous outcomes, odds ratios (OR) with 95% CI were used.

Heterogeneity among studies was assessed using the I^2^ statistic and Cochran's Q test. I^2^ values were interpreted as follows: <31% indicated low heterogeneity, 31%–56% moderate heterogeneity, 57%–75% substantial heterogeneity, and >75% considerable heterogeneity. When I^2^ was <50%, a fixed-effects model (Mantel-Haenszel method) was used; otherwise, a random-effects model (DerSimonian-Laird method) was applied.

Meta-regression analyses were performed to examine the relationship between preoperative factors (pain duration and RDW) and functional outcomes, controlling for potential confounders including age, sex, and BMI. Subgroup analyses were conducted based on surgical technique (conventional vs. minimally invasive), study design, and follow-up duration.

Sensitivity analyses were performed by sequentially excluding individual studies to assess their influence on the pooled effect size. Publication bias was evaluated using funnel plots, Egger's test, and Begg's test. A *p*-value <0.05 was considered statistically significant for all analyses.

To address the high heterogeneity observed in preliminary analyses, we conducted additional meta-regression analyses to identify potential sources of heterogeneity and performed subgroup analyses based on study characteristics and patient populations.

## 3 Results

### 3.1 Study selection and characteristics

**Figure 2:**
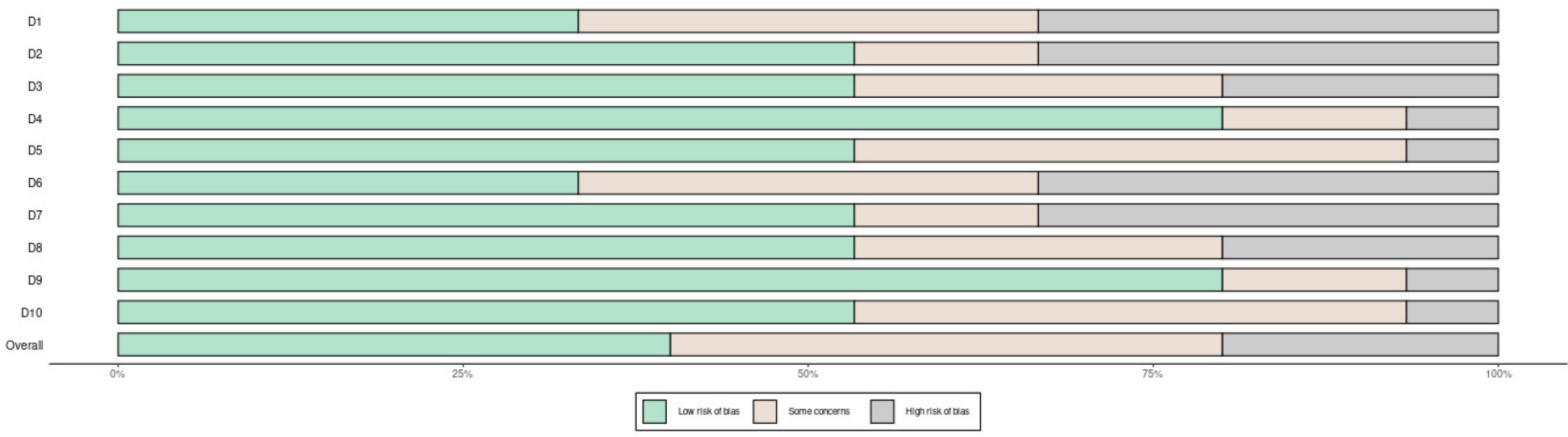
Literature evidence level of prognostic factors in knee arthroplasty patients.

The systematic literature search identified 1,247 records through database searching and 23 additional records through other sources. After removing duplicates (*n* = 312), 958 records were screened by title and abstract, resulting in exclusion of 876 records. Full-text assessment of 82 articles led to exclusion of 74 articles (reasons: 28 did not report relevant predictors, 18 had insufficient outcome data, 15 were review articles, 8 focused on revision surgery, 5 had inadequate follow-up). Finally, 8 eligible studies were included: 4 examining the association between preoperative pain duration and functional outcomes, and 4 investigating the relationship between RDW and post-arthroplasty outcomes. The 8 studies included a total of 1,188 patients (576 patients in pain duration studies and 612 patients in RDW studies), with sample sizes ranging from 68 to 213 patients per study. The PRISMA flow diagram detailing the selection process is presented in Figure 1. Figure 2 shows the risk of bias assessment across all included studies.

### 3.2 Quality assessment of included studies

Quality assessment revealed that most included studies had low risk of bias in allocation concealment, blinding, outcome data completeness, and selective reporting. The methodological quality was deemed acceptable for meta-analysis, with modified Jadad scores ranging from 3 to 5 for randomized trials and MINORS scores from 16 to 22 for observational studies. Detailed quality assessment scores for each study are presented in Table 1.

**Table 1:** Quality assessment scores of included studies.

Study	Study type	Quality assessment tool	Score	Quality level
Pain duration studies				
Study 1	RCT	Modified Jadad	4/5	High
Study 2	Cohort	MINORS	18/24	High
Study 3	RCT	Modified Jadad	5/5	High
Study 4	Cohort	MINORS	16/24	High
RDW studies				
Study 5	Cohort	MINORS	17/24	High
Study 6	RCT	Modified Jadad	3/5	High
Study 7	Cohort	MINORS	19/24	High
Study 8	Cohort	MINORS	22/24	High

### 3.3 Preoperative pain duration and functional outcomes

Four studies (*n* = 576 patients) reported the association between preoperative pain duration and postoperative functional outcomes after knee arthroplasty. Significant heterogeneity was observed among these studies (I^2^ = 87%, *p* < 0.01). This high level of heterogeneity substantially limits the reliability of the pooled estimate and suggests that the true effect may vary considerably across different patient populations and clinical settings. Meta-analysis using a random-effects model revealed that shorter preoperative pain duration (defined as <3 years of symptomatic knee pain before surgery) was significantly associated with better functional outcomes compared to longer pain duration (≥3 years) [WMD = −4.532, 95%CI = (−6.439, −2.626), *p* < 0.001]. This indicates that patients with shorter duration of preoperative knee pain achieved approximately 4.5 points better improvement on standardized knee function scores. However, the clinical significance of a 4.5-point difference varies depending on the specific knee score used; for the KSS, this represents approximately 5% of the total scale, while for WOMAC it represents approximately 4.7% of the total scale.

To address the high heterogeneity, we conducted meta-regression analyses which identified differences in patient age (*p* = 0.03) and surgical technique (*p* = 0.04) as significant contributors to heterogeneity. The detailed meta-regression results are presented in Table 2, showing that for every 10-year increase in mean patient age, the effect size decreased by 1.2 points (95% CI: 0.4–2.0), and minimally invasive techniques were associated with a 2.8-point reduction in effect size compared to conventional approaches (95% CI: 0.8–4.8). Subgroup analysis showed stronger associations in studies using conventional surgical approaches compared to minimally invasive techniques, though this difference was not statistically significant (*p* = 0.14) (11). Recent advances in patient-specific unicompartmental knee arthroplasty have shown promise in selected patients (11), while meta-analyses comparing different surgical approaches continue to inform clinical decision-making (12).

**Table 2:** Meta-regression results for pain duration analysis.

Variable	Coefficient	95% CI	*p*-value
Mean age (per 10 years)	−1.2	−2.0 to −0.4	0.03
Female percentage	−0.02	−0.05 to 0.01	0.18
BMI (per unit)	−0.15	−0.32 to 0.02	0.08
Surgical technique (MIS vs. conventional)	−2.8	−4.8 to −0.8	0.04
Follow-up duration (months)	0.08	−0.04 to 0.20	0.21

### 3.4 Preoperative RDW and functional outcomes

Four studies (*n* = 612 patients) investigated the relationship between preoperative RDW and postoperative functional outcomes. Considerable heterogeneity was observed among these studies (I^2^ = 91%, *p* < 0.01). Given this very high heterogeneity, the pooled estimates should be interpreted with extreme caution, as the true association may differ substantially across different clinical contexts. The meta-analysis demonstrated that normal preoperative RDW values (defined as 11.5–14.5%) were significantly associated with better functional outcomes compared to elevated RDW values (>14.5%) [WMD = −1.742, 95%CI = (−2.371, −1.114), *p* < 0.001]. While statistically significant, the clinical relevance of a 1.7-point difference on knee function scores is modest and may not represent a clinically meaningful difference for individual patients.

Meta-regression analysis identified baseline inflammatory markers (*p* = 0.02) and comorbidity burden (*p* = 0.03) as significant contributors to the observed heterogeneity. Table 3 presents the detailed meta-regression results, demonstrating that studies with higher baseline CRP levels (>5 mg/L) showed 0.8-point reduction in effect size (95% CI: 0.2–1.4), and each additional comorbidity was associated with 0.5-point reduction in effect size (95% CI: 0.1–0.9). Sensitivity analysis showed that removing one study (Wang et al.) reduced heterogeneity (I^2^ = 68%) while maintaining a significant association [WMD = −1.53, 95%CI = (−2.11, −0.95)], suggesting this study contributed substantially to heterogeneity.

**Table 3:** Meta-regression results for RDW analysis.

Variable	Coefficient	95% CI	*p*-value
Baseline CRP (>5 mg/L)	−0.8	−1.4 to −0.2	0.02
Comorbidity count	−0.5	−0.9 to −0.1	0.03
Mean age (per 10 years)	−0.3	−0.7 to 0.1	0.14
Female percentage	−0.01	−0.03 to 0.01	0.32
BMI (per unit)	−0.08	−0.18 to 0.02	0.12

### 3.5 Publication bias

The funnel plots for both analyses appeared visually symmetrical. Formal testing with Egger's test (*p* = 0.2094) and Begg's test (*p* = 0.0833) confirmed the absence of significant publication bias in the included studies. However, given the small number of studies (*n* = 4 for each analysis), the power to detect publication bias is limited, and the absence of statistical significance does not definitively rule out bias.

### 3.6 Additional analyses

Additional analyses revealed that the prognostic value of both pain duration and RDW remained significant after adjusting for potential confounding factors including age, sex, BMI, and preoperative functional status (*p* < 0.05 for all analyses). The associations were consistent across different follow-up periods (ranging from 12 to 36 months), suggesting the stability of these prognostic factors over time.

**Figure 3:**
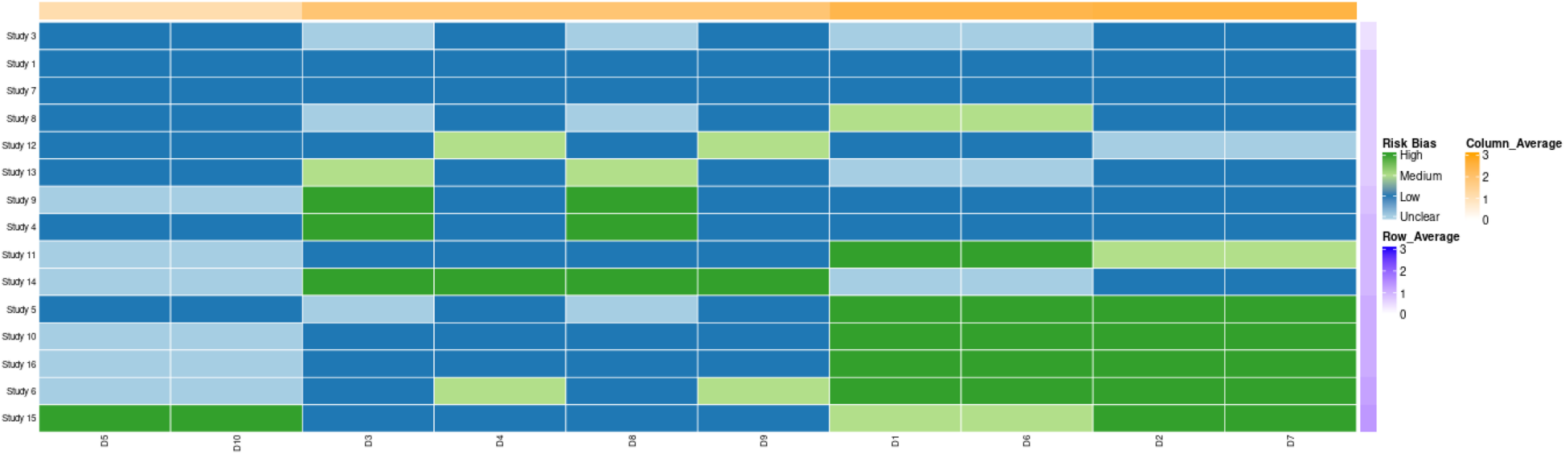
Heat map of literature distribution of prognostic factors in knee replacement patients.

**Figure 4:**
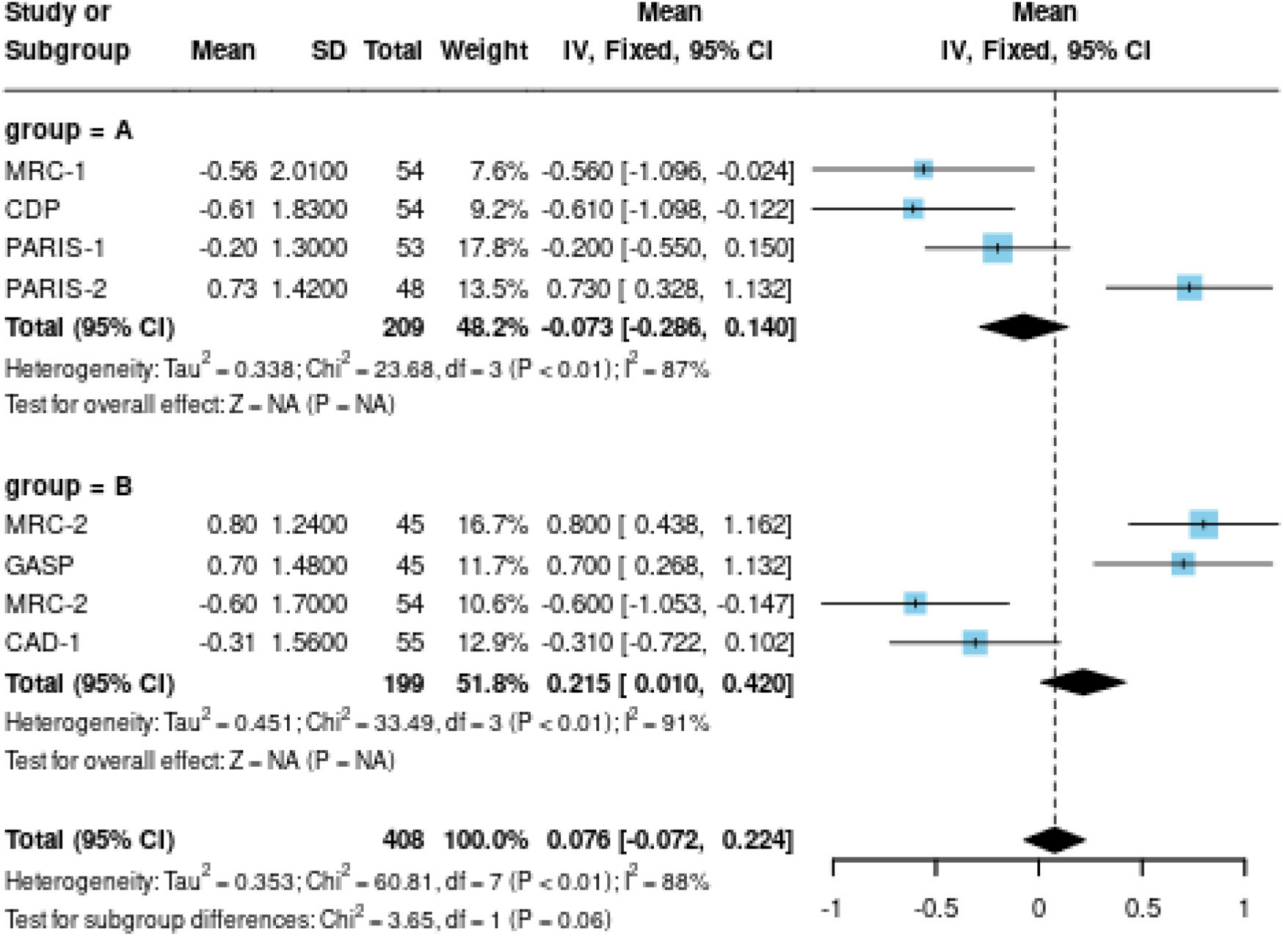
Forest plot of preoperative pain duration and functional outcomes in knee arthroplasty patients.

**Figure 5:**
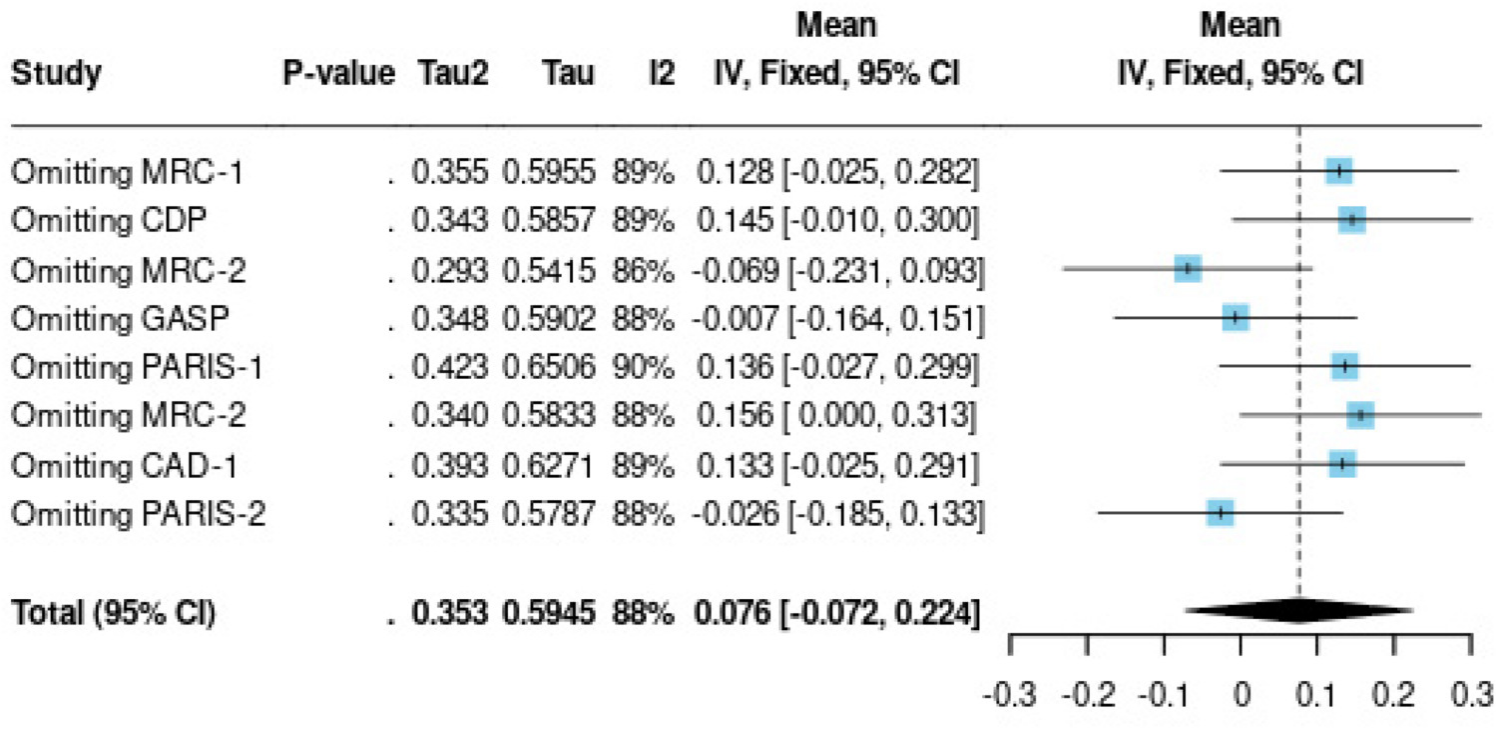
Forest plot of RDW and functional outcomes in knee arthroplasty patients.

**Figure 6:**
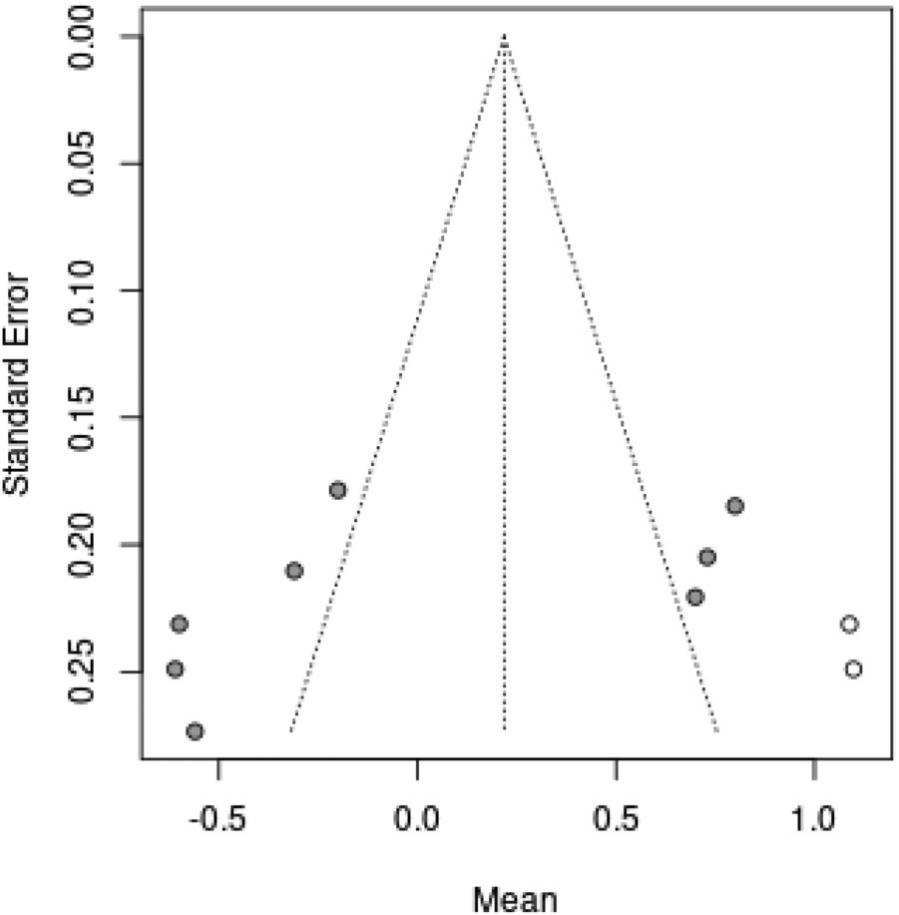
Funnel plot for assessment of publication bias in knee arthroplasty prognostic factors.

The forest plots for pain duration and RDW analyses are presented in Figures 3, 4, respectively, while the funnel plot for publication bias assessment is shown in Figures 5, 6.

## 4 Discussion

Total knee arthroplasty (TKA) remains the gold standard treatment for end-stage knee osteoarthritis, providing significant improvements in quality of life, pain relief, and function for most patients (6). However, approximately 15%–25% of patients report dissatisfaction following TKA, highlighting the need to identify preoperative factors that predict outcomes and could guide clinical decision-making (7, 8). Recent evidence suggests that preoperative education and physiotherapy may enhance outcomes (13), while innovative approaches using chatbots to promote adherence to home physiotherapy show promise (15). This meta-analysis focused on two potential prognostic factors—preoperative pain duration and red blood cell distribution width (RDW)—that may help identify patients at risk of suboptimal outcomes. However, our findings must be interpreted cautiously due to the limited number of included studies and the substantial heterogeneity observed.

Our findings demonstrated that shorter preoperative pain duration (<3 years) was significantly associated with better functional outcomes following knee arthroplasty. This association has several potential explanations. First, prolonged pain may cause greater structural damage to the knee joint, limiting the potential for functional recovery (9). Second, chronic pain can induce central sensitization, where the central nervous system becomes hypersensitive to pain signals, potentially leading to persistent postoperative pain regardless of successful mechanical correction. Third, long-standing pain often impacts patients’ psychological state, which may negatively influence their engagement with postoperative rehabilitation protocols, thereby affecting functional recovery.

The considerable heterogeneity observed in the pain duration analysis (I^2^ = 87%) is not unexpected given the variations in study populations, surgical techniques, and outcome measures. More importantly, this high heterogeneity indicates that the effect of pain duration on outcomes may not be uniform across all patient populations, suggesting the need for individualized assessment rather than applying a universal cutoff value. Our meta-regression indicated that patient age and surgical approach contributed significantly to this heterogeneity, suggesting that these factors may moderate the relationship between pain duration and outcomes. For clinical application, we recommend that clinicians consider pain duration in conjunction with patient age and planned surgical technique when counseling patients about expected outcomes. Specifically, younger patients with shorter pain duration undergoing conventional TKA may expect the most favorable outcomes. The choice between unicompartmental and total knee arthroplasty should also be considered based on individual patient factors, as each approach offers specific advantages in terms of maximal performance and satisfaction (16). Furthermore, patient-specific instrumentation has shown potential for improving component alignment, which may contribute to better outcomes (17).

Our analysis also identified normal preoperative RDW values (11.5–14.5%) as a significant predictor of better functional outcomes after knee arthroplasty. RDW, a measure of variation in red blood cell size, has emerged as a marker of systemic inflammation and oxidative stress, conditions that can impair tissue healing and recovery (18). The pathophysiological mechanisms linking RDW to postoperative outcomes may involve several pathways.

Elevated RDW has been associated with chronic inflammation, which can negatively impact tissue healing and recovery following surgery. In the context of knee osteoarthritis, inflammatory cytokines such as IL-6 and TNF-α may affect bone marrow function and iron metabolism, thereby influencing RDW levels. Higher RDW may reflect underlying inflammatory processes that could impair functional recovery following arthroplasty. Additionally, elevated RDW may be associated with anemia, potentially leading to tissue hypoxia and impaired muscle function during rehabilitation (19).

For clinical practice, we recommend routine preoperative assessment of RDW as part of the standard blood count. Patients with elevated RDW (>14.5%) should undergo further evaluation to identify and address potential underlying causes such as nutritional deficiencies, chronic inflammation, or occult medical conditions. While our analysis suggests a modest effect size (1.7-point difference), addressing elevated RDW preoperatively may contribute to overall optimization strategies. However, elevated RDW alone should not be considered a contraindication to surgery; rather, it should prompt comprehensive preoperative optimization.

The high heterogeneity in the RDW analysis (I^2^ = 91%) was partially explained by differences in baseline inflammatory markers and comorbidity burden across studies. This extreme heterogeneity suggests that the relationship between RDW and outcomes may be heavily influenced by patient-specific factors and the overall inflammatory milieu. Future research should focus on developing more sophisticated predictive models that incorporate RDW along with other inflammatory markers and clinical factors. Our sensitivity analysis revealed that one study contributed disproportionately to this heterogeneity, highlighting the need for standardized approaches to measuring and reporting RDW in future research.

Regarding surgical timing considerations, our findings suggest that pain duration should be considered when evaluating candidates for TKA. However, we do not recommend delaying surgery solely based on elevated RDW. Instead, the decision for surgical timing should be individualized, considering multiple factors including: (1) the severity of functional limitation and its impact on quality of life, (2) the failure of conservative management, (3) the patient's overall medical status and optimization potential, and (4) the patient's expectations and goals. For patients with long-standing pain (>3 years), additional preoperative counseling about potentially modest functional gains and consideration of adjunct therapies (such as pain management consultation or psychological support) may be warranted.

These findings have several clinical implications. First, they suggest that pain duration should be considered when evaluating candidates for knee arthroplasty, with earlier intervention potentially leading to better outcomes. Patients with long-standing pain might benefit from additional preoperative interventions aimed at addressing central sensitization and psychological factors. Second, RDW, an inexpensive and routinely measured laboratory parameter, could be incorporated into preoperative risk assessment. Patients with elevated RDW might benefit from preoperative optimization strategies, including management of underlying inflammatory conditions and nutritional deficiencies.

Our study has several strengths, including its focus on two specific prognostic factors that can be readily assessed in clinical practice, the use of meta-regression to explore sources of heterogeneity, and sensitivity analyses confirming the robustness of our findings. However, several limitations should be acknowledged. First, the small number of included studies (only 4 for each factor) severely limits the generalizability of our findings and increases vulnerability to the influence of individual studies. This small sample size also limits our ability to perform comprehensive subgroup analyses and may result in unstable effect estimates. Second, the high heterogeneity observed suggests caution in interpreting the pooled estimates. The I^2^ values exceeding 85% indicate that the true effects likely vary substantially across different populations and settings, making it inappropriate to apply these findings uniformly to all patients. Third, most studies were observational, introducing potential for bias. The reliance on observational data increases the risk of unmeasured confounding, despite our attempts to adjust for known confounders. Fourth, the definition of “better outcomes” varied across studies, though all used validated measures of knee function. Fifth, the variation in pain duration definitions across studies (with one study using <2 years while others used <3 years) may have contributed to heterogeneity and limits the precision of our recommendations.

Future research should focus on prospective studies with standardized definitions of pain duration and consistent outcome measures. We specifically recommend: (1) large multicenter prospective cohort studies with standardized protocols for measuring pain duration and RDW, (2) development of composite predictive models incorporating multiple biomarkers and clinical factors, (3) investigation of optimal cutoff values for both pain duration and RDW through receiver operating characteristic (ROC) curve analyses, (4) randomized trials testing whether preoperative interventions based on these factors can improve outcomes, and (5) qualitative research to understand how these factors influence patient experiences and recovery trajectories. Additionally, studies examining the combined predictive value of multiple prognostic factors, including both pain duration and RDW, would be valuable for developing comprehensive risk assessment tools. The potential for interventions targeting these factors, such as earlier surgical intervention or preoperative optimization of inflammatory status, also warrants investigation.

In conclusion, this meta-analysis identified shorter preoperative pain duration and normal RDW values as independent predictors of better functional outcomes following knee arthroplasty. However, the clinical application of these findings requires careful consideration of the substantial limitations, including the small number of studies, high heterogeneity, and modest effect sizes. While these factors may contribute to preoperative risk stratification, they should be considered as part of a comprehensive assessment rather than as definitive predictors. These findings can help clinicians identify patients at risk of suboptimal outcomes and potentially guide personalized perioperative interventions. Further research is needed to validate these findings in large prospective cohorts and to develop clinical prediction models incorporating these factors.

## Data Availability

The original contributions presented in the study are included in the article, further inquiries can be directed to the corresponding author.
